# Investigation of the Importance of Knee Position during Femoral Tunnel Reaming; Figure 4 versus Hyperflexion

**DOI:** 10.5704/MOJ.2207.013

**Published:** 2022-07

**Authors:** A Kose, MS Ayas, MC Turgut, ON Altay

**Affiliations:** 1 Department of Orthopedics and Traumatology, Erzurum Bolge Egitim ve Arastirma Hastanesi, Erzurum, Turkey; 2 Department of Orthopedics and Traumatology, Karadeniz Technical University, Trabzon, Turkey

**Keywords:** anterior cruciate ligament, reconstruction, femoral tunnel, figure 4, hyperflexion

## Abstract

**Introduction:**

We aimed to compare whether the visualisation provided by arthroscopic hyperflexion and Figure 4 has an effect on femoral tunnel placement in patients undergoing single bundle Anterior Cruciate Ligament Reconstruction (ACLR).

**Material and methods:**

We retrospectively evaluated 93 patients who underwent single-band ACLR for Anterior Cruciate Ligament (ACL) injury between 2016 and 2019. Eighty patients met the inclusion criteria with a minimum follow-up of 12 months. We divided the patients into Group 1 (figure 4) and Group 2 (hyperflexion). We analysed the demographic, radiological and functional outcomes of the patients. The functional Lysholm score, operative time, radiological Quadrant method (% proximal-distal and % anterior-posterior) measurements, tunnel lengths, axial and coronal plane angles, and iatrogenic chondral injury in the medial femoral condyle were evaluated.

**Results:**

Iatrogenic chondral injury developed in the medial femoral condyle in a total of seven patients in both groups: one patient in group 1 (Figure 4) and six patients in group 2 (Hyperflexion). Although statistically insignificant, iatrogenic medial femoral condyle damage was less in group 1. The statistical analysis between surgical operation time (p = 0.046) and tunnel lengths (p = 0.042) was significant.

**Conclusion:**

The position of figure 4 provides visualisation of lateral intercondylar notch better than hyperflexion. In the reaming stage, the medial femoral condyle is less damaged in group 1 (Figure 4). In ACLR, which has a long learning curve, short surgery time is seen as an important advantage for surgeons who have just started doing ACLR. We think that it can be used as an alternative method to hyperflexion in the learning process and maybe shorten the learning curve process.

## Introduction

Intra-articular visualisation plays an important role in the success of arthroscopic surgical treatments^1,2^. The search for new surgical techniques and materials is still ongoing to increase visualisation and 3D orientation^3-6^. Methods such as; accessory portal usage, camera usage at 30° or 70° angle, increased or decreased joint flexion, varus or valgus torque, increase or decrease in internal rotation, a computerised navigation application, intra-operative navigation application, traction applications and fluoroscopic control during surgery are frequently used in arthroscopic practice^2,7,8^. The importance of visualisation increases even more in cases such as meniscus repair, meniscectomy, microfracture, and ligament reconstructions^1^. Particularly in primary Anterior Cruciate Ligament Reconstruction, the creation of a femoral tunnel is one of the challenging steps for surgeons^9^. To overcome this difficulty, it is necessary to provide a clear visualisation of the lateral femoral intercondylar notch. For this purpose, figure 4 (described as figure 9 in some publications) and hyperflexion of the different angle of the tunnel formation has been reported^1,8,10^. According to the direction of the tunnel reaming; Inside-out (transtibial, transportal) and outside-in methods are described. In the transportal method, inside-out reaming is frequently preferred^6,11,12^. In this method, rymirization should be done in hyperflexion. No matter how intra-articular debridement is performed, the area entering the visual field decreases in the lateral intercondylar notch as knee flexion increases^8^. In particular, the posterior condylar border not entering the image area clearly may result in incorrect placement of the guidewire. Posterior wall fracture may develop if the distance of the tunnel, especially the posterior condylar is not adjusted properly^6,11^.

In reviewing the literature of ACL injury, the ideal femoral tunnel position is still controversial despite the defined bone (Bifurcate ridge, Resident ridge) and soft tissue landmarks (remnant footprint, lateral meniscus posterior horn) and surgical methods. Tunnel insertions and tunnel positions create the most important stage of treatment. In ACLR, the first and the most important step is to create a tunnel using remnant footprints and anatomical landmarks. The Selected graft tunnel formed is placed at the proper angle and tension. Then fixation is applied with suitable fixing materials. The main goal of treatment is the anatomical presence of the graft and it’s functional mimicking of the ligament^12-16^. It is known that malposition of the femoral tunnel is one of the most important causes of failure and rupture in ACLR. Possible minor errors in tunnel placement may cause elongation of graft, incompatibility in graft tension, and rerupture^8,10,17^.

The effect of hyperflexion and figure 4 positions on arthroscopic visualisation is known^1,7^. In studies, evaluation of posterolateral bundle tension in Figure 4 position and lateral meniscus posteromedial root tears is emphasised^10,18^. However, in the literature, we did not find a report showing whether it affects femoral tunnel placement. Therefore, in our study, we aimed to compare whether the visualisation provided by arthroscopic hyperflexion and four positions has an effect on femoral tunnel placement in patients undergoing ACLR.

## Materials and Methods

We evaluated patients in whom we performed single bundle ACLR due to Anterior Cruciate Ligament (ACL) injury. We analysed the demographic, radiological, and functional results of the patients. The femoral tunnel of these patients was formed in figure 4 (Group 1) or positions in hyperflexion (Group 2) (confirming from arthroscopic video recordings). We determined the treatment according to the order of hospitalization of the patients for surgery. Surgical procedure was performed in the hyperflexion position (Group 2) for the patients who had an odd number (eg. 1,3,5,7, ...) in the list during the hospitalisation procedure. Surgical procedure was performed in the figure 4 position (Group 1) for the patients who had an even number (eg. 2, 4, 6, 8, ...) in the list during the hospitalisation procedure. We evaluated 80 patients retrospectively between January 2016 and March 2019. We included patients in this study with isolated ACL rupture who underwent single band reconstruction with an autogenous hamstring graft (gracilis and semitendinosus tendons). We included the patients who received 3D CT images after the operation for tunnel placement, underwent a minimum one-year follow-up, patients with graft fixation using the endobutton suspension system. We excluded patients who had undergone different surgical procedures in the same knee, underwent revision surgery, accompanying ligament injury, patients with deformity and fracture that will affect axial alignment in the same limb, patients with stage 2 and above osteoarthritis findings, patients without 3D CT after surgery, patients with immature skeletal systems, patient’s undergoing bilateral ACLR and those with a follow-up period of less than one year. Our institutional review board approved this study.

We checked the data of 93 patients who underwent ACLR within the specified date range. We excluded the following patients; six patients who were operated on due to concomitant ligament injuries, two patients who underwent bone tendon bone revision surgery, three patients who did not receive 3D CT after surgery, and two patients who did not want to participate in the study. Eighty patients who met the specified criteria were included in the study. We divided the patients into two groups: The femoral tunnel entry site created in Figure 4 (Group 1:40 patients) and the femoral tunnel entry site created in Hyperflexion (Group 2:40 patients).

We evaluated the demographic, radiological, and functional results of the patients for statistical comparison. Table I shows the age, side of surgery, gender, time from primary injury to operation, body mass index (BMI), and graft thickness. In Table II, we evaluated respectively; accompanying surgical procedures (meniscectomy, meniscus repair, microfracture), follow-up times, functional Lysholm score, duration of surgery, radiological Quadrant method 5 (% proximal-distal and % anterior-posterior) measurements, tunnel lengths, axial and coronal plan angles.

**Table I:** Demographic, functional and radiological data of patients (Mean, minimum, maximum, standard deviation)

	Group 1	SD	Group 2	SD
Age	29 (18-44)	6.87	29 (18-44)	6.54
Time from the moment of trauma to the operation (months)	9 (1-36)	10.31	8 (1-35)	9.51
Operation side (Right / left)	24/16		22/18	
Gender (Male / Female)	30/10		32/8	
BMI (kg / m2)	26 (20-31)	3.26	27 (22-31)	2.29
Graft Thickness (mm)	8.3 (8-10)	0.57	8.4 (7-10)	0.82
Lysholm score	89.75 (75-100)	8.02	87 (60-100)	10.3
Tunnel Length (mm)	39.7 (30-45)	4.78	36.7 (30-45)	4.25
Operation Time (minutes)	58 (45-75)	7.18	63.7 (45-80)	9.78
Follow-up time (months)	27.7 (12-60)	13.7	27.9 (14-58)	9.78
Coronal Tunnel angle	38 (35-42)	1.92	39 (35-48)	3.74
Axial Tunnel angle	39 (35-46)	2.70	38 (29-44)	3.15
Quadrant Method (% Anterior-Posterior)	32 (26-38)	2.93	32 (29-36)	2.29
Quadrant Method (%Proximal-Distal)	33 (27-37)	2.83	32 (26-36)	2.61
Concomitant operation				
No	22		20	
Partial Meniscectomy	4		7	
Meniscus repair	10		10	
Microfracture	4		3	
Complication				
Medial Femoral Chondral injury	1		6	

**Table II:** Comparison of Lysholm score, tunnel length, operation time and radiological data in Groups 1 and 2 with Student's t test

	Independent Samples Test	Sig.(2-tailed)
Lysholm score	0,855	0,398
Tunnel Length (mm)	2,061	0,046
Operation Time (minutes)	-2,100	0,042
Coronal Tunnel angle	-1,010	0,319
Axial Tunnel angle	0,646	0,522
Quadrant Method (%Anterior-Posterior)	-0,240	0,812
Quadrant Method (%Proximal-Distal)	0,928	0,360

**Fig 1:**
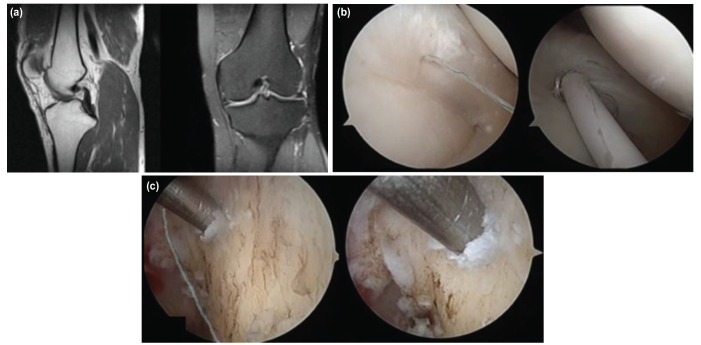
The 28-year-old male patient has ACL injury and meniscus tear in his left knee, (a) Sagittal and axial section MR image, (b) repair of the medial meniscus tear with all inside suture, (c) Placement of the guidewire, view of the distance to the inferior and posterior cortex (blue line).

**Fig 2:**
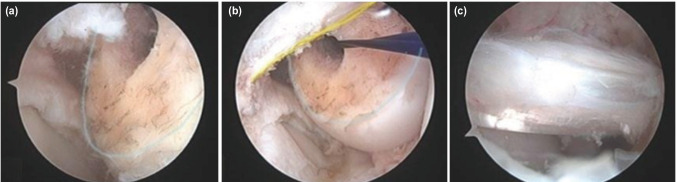
(a, b) in figure 4, the lateral meniscus and clear visualisation of the tunnel created (blue and yellow line), (c) view of the graft placement.

**Fig 3:**
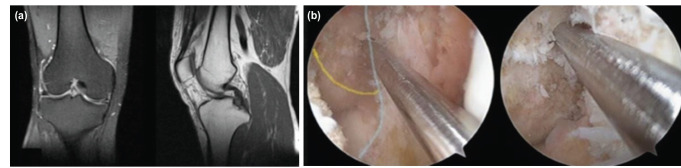
The 32-year-old male patient has ACL injury in the right knee, (a) Sagittal and axial section MR image, (b) Placement of the guidewire, the appearance of the distance to the inferior and posterior cortex, preventing PCL from the visualisation of the posterior cortex (blue line).

**Fig 4:**
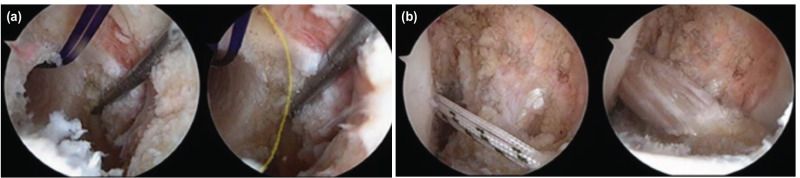
(a) Tunnel placement, control of the distance of the tunnel from the inferior and posterior cortex with the examination probe (blue and yellow line), (b) in hyperflexion, the posterior border is not visible and the appearance of the graft placement.

The 3D CT taken on the first post-operative day was evaluated together with the radiologist. Quadrant method measurements (% proximal-distal and % anterior-posterior), tunnel lengths, axial, and coronal plane angles were evaluated for all patients. We have received the surgery under video images in digital format. We evaluated the surgical procedures we performed retrospectively. We evaluated the operation times, concomitant operations, condylar damage, or other complications that occurred during the operation, visualisation provided by figure 4 or hyperflexion positions from the surgery records (Fig. 1-4). This evaluation was performed by another surgeon who did not participate in the surgical procedure.

All surgical procedures were performed by a single surgeon. A tourniquet was applied before surgery. An arthroscopic evaluation and examination made initially before the graft was taken. We recorded all arthroscopy applications in the digital environment, and we did not use an accessory arthroscopic portal (Far medial or near patellar). A single portal was used as there was less damage to the capsule and no difficulty in guiding the guidewire. We set the medial portal at an angle where we can directly access all intra-articular structures with the gray intracet. The medial portal was set at an angle and position to perform all surgical procedures and we applied the necessary Hoffa debridement and radiofrequency cauterisation. In necessary cases, we performed a meniscectomy, meniscus repair and microfracture. The femoral and tibial footprints were used as anatomic centres in tunnel formation, and we quadrupled the autologous Hamstring tendons. A rigid guidewire was used for the femoral tunnel entry site and its placement. An offset guide was not used when placing the guidewire and we performed the process with a freehand. We placed the guidewire in the hyperflexion or figure at the 4 positions (Fig. 1, 2). In some patients we had to confirm whether the guidewire passed through the anatomical footprint in hyperflexion (Fig. 3, 4). In particular we had to control the distance from the posterior cortex. For this reason, we have made some position changes to increase visualisation. We reduced knee flexion to 90° and brought the camera to the same portal (medial portal) with the guidewire and checked the accuracy of the first entry. We then switched to a hyperflexion position. By advancing the guidewire, we made it out of the lateral cortex. In Figure 4 position, we did not change the portal and position. We applied the guidewire and made rymirization over the guidewire. We performed the rymirization in its position in both techniques. We detected the graft on the femoral bone with the XO Button [ConMed Linvatec, Inc. Largo, FL] suspension system. We detected the tibia bone with both bioabsorbable interference screw and stapler.

The statistical analysis was performed using SPSS Version 22.0 statistical analysis software. The data are presented as the number, percentage, average, and standard deviation. The compliance of the data to the normal distribution was analysed with the Kolmogorov Smirnov Test. Student T-Test was used to analyse the data. Statistical significance value was taken as p <.05.

## Results

The study included 62 men and 18 women and overall 80 patients were included. The average age was 29 yrs (18-44) [Group 1 (Figure 4); 29±6.87 (18-44), Group 2 (Hyperflexion); 29±6.54 (18-44)]. Surgery was performed on the right knee in 46 patients and the left knee in 34 patients. We only performed ACLR on 42 patients. In addition to ACLR, we performed partial meniscectomy in 11 patients, repair of the meniscus in 20 patients, and microfracture in 7 patients. The demographic data of the patients in group 1 (Figure 4) and group 2 (Hyperflexion) are presented in Table I.

None of the patients developed early or late infections. Iatrogenic chondral injury developed in the medial femoral condyle in 7 patients, 1 in group 1 (Figure 4) and 6 in group 2 (Hyperflexion). No other intra-operative complications developed. We watched the entire operation video recordings. Visualisation was evaluated in figure 4 and hyperflexion positions. While the camera was in the lateral portal, we evaluated the posterior cortex border of the lateral femoral condyle and whether the posterior root of the lateral meniscus was observed. In group 1 (Figure 4), we obtained a complete image in all patients. However, we evaluated that we could not obtain a complete image of 15 patients in group 2 (Hyperflexion). We evaluated; Lysholm score 89.75±8.02 (75-100) in group 1 (Figure 4), 87±10.3 (60-100) in group 2 (Hyperflexion), coronal tunnel angle (degree) 38±1.92 (3542) in group 1 (Figure 4), 39±3.74 (35-48) in group 2 (Hyperflexion), axial tunnel angle (degrees) 39±2.70 (35-46) in group 1 (Figure 4), 38±3.15 (29-44) in group 2 (Hyperflexion), percentage of Quadrant Method (% Anterior-Posterior) 32±2.93 (26-38) in group 1 (Figure 4), 32±2.29 (29-36) in group 2 (Hyperflexion), percent of Quadrant Method (% Proximal-Distal) 33±2.83 (27-37) in group 1 (Figure 4), 32±2.61 (26-36) in group 2 (Hyperflexion), respectively. We evaluated the average of tunnel length as 39.7±4.78 (30-45) mm in group 1 (Figure 4) and 36.7±4.25 (30-45) mm in group 2 (Hyperflexion). The tunnel length means was evaluated higher in group 1 (Figure 4) than in group 2 (Hyperflexion). This rate was statistical difference between both groups (p=.046). The mean operation time was 58±7.18 (45-75) minutes in group 1 (Figure 4) and 63.7±9.78 (45-80) minutes in group 2 (Hyperflexion). In group 1 (Figure 4), the mean duration of surgery was shorter than group 2 (Hyperflexion), and this rate was found statistical difference (p=.042). There was no statistical difference between both groups (p>.05) in the Lysholm score mean, in coronal tunnel angle means, axial tunnel angle means, in Quadrant method anterior-posterior percentage mean, and Quadrant method proximal-distal percentage means (Table II). Procedures in addition to ACLR (partial meniscectomy, meniscus repair, microfracture) have extended the average operation time. However, there was no statistical difference between the mean operative time of patients who underwent additional surgery in both groups and only those who underwent ACLR (p>.05).

## Discussion

The most important finding of the study was the figure 4 position provides longer tunnel length and shortens the surgical time. In addition, the medial femoral condyle is less damaged. Radiological and functional results of both methods were found to be similar. Although the guide anatomical landmark and remnant footprints are used in primary ACLR, many issues related to tunnel placement are still controversial. In single band ACLR, the anatomy of the entrance site, interosseous spatial placement of the tunnel, angle of entry of the graft into the tunnel, exit point in the lateral cortex, the geometry of the tunnel mouth (round-oval), tunnel diameter, minimum tunnel length for graft osteointegration, the distance of the tunnel from the posterior cortex are among the controversial topics^10,18^. There are many types of research on the use of anatomical landmarks, use of cameras at different angles (30° or 70° camera), use of femoral offset guide, midbundle tunnel entrance, the geometry of the tunnel entrances (oval-round), angle of entry of the graft into the tunnel, in primary ACL^5,11,14,18^. Current studies focus on the individuality of reconstruction and preoperative planning^17^. But the ideal placement of the tunnel is still controversial^17^. Therefore, to minimise the number of cases of revision ACLR, treatment should not be left to chance in primary ACLR.

Figure 4 position is a hyperflexion position with increased internal rotation and varus. The importance of evaluation of the tension of the posterolateral bundle of ACL is emphasised^1^. Figure 4 shows the lateral intercondylar notch, anatomical landmarks, posterior cortex, inferior and posterior cartilage border, anterior surface of the lateral meniscus better than hyperflexion^1,8^. However, increasing varus torque may make it difficult to apply the transportal of guidewire and reamer. As a result, medial femoral condyle or meniscus damage may occur. Also, in the figure 4 position, the exit point of the guidewire is below the operating table. Despite adequate Hoffa, soft tissue, and remnant tissue debridement, direct visualisation of the anatomical borders may be limited in the hyperflexion position. The Posterior Cruciate ligament and synovial tissues fill the intercondylar notch volumetrically and lateral condylar can prevent the border from appearing clearly^19^. We evaluated the visualisation of the lateral femoral condyle inner surface from the operation video recordings. In Group 1, we obtained a complete image in all patients. However, we could not obtain a clear view of the posterior condylar border in 15 (37.5%) patients in group 2. Therefore, we had to do some manoeuvres to ensure the distance from the posterior condylar border and optimal footprint centralisation. We reduced the flexion angle to 90°. By taking the camera into the medial portal, we evaluated the entrance of the guidewire. Maybe we had to repeat the same process several times. As a result, the average surgical time in group 2 may be prolonged (Table I-II).

Many different techniques and methods have been defined in ACLR to minimise complications and provide optimal tunnel placement and length^20,21^. Cadaveric and clinical studies have been conducted to standardise treatment and to optimise controversial issues^20,21^. In these studies, the knee flexion angle for rigid or flexible rymirization has been suggested to be 110° - 125°. It has been reported that rigid pins do not approach the posterior cortex compared to flexible pins in the selection of guidewire used for rymirization. However, creating a tunnel with 110° knee flexion and a 10mm rymir puts the posterior cortex at risk. It was stated that the posterior cortex blow-out fracture was not observed while applying curved rymirization. In rigid rymirization, 2 blow-out fractures were observed at 90° flexion and 3 at 110° flexion^21^. It was reported that blow-out occurred in 2 patients in the series of 50 cases^21^. In our study, we performed rymirization in both groups over a rigid guidewire. However, we performed rymirization in 100° - 110° flexion in group 1 and maximum hyperflexion in group 2. No patients developed blow-out fractures in either group. The angle of flexion during surgery is affected by many factors^6,8,17^. Individual factors such as muscle atrophy, person's sporty lifestyle, leg length, as well as tourniquet thickness are some of them^6,8,17^. It is therefore difficult to standardise and maintain the knee flexion angle during surgery. Also, it complicates patient randomisation. Although the length of the tunnel increased as the flexion angle is increased, the optimal tunnel length is still controversial. Minimum tunnel length is recommended to be >25mm. It provides optimisation of the anatomical footprint of rymirization in flexion over 125°^21^. In the study of Kosy *et al*, the average tunnel length with flexible rymir was 37.8±3.7mm, while it was 35±4.4mm with rigid rymir^6^. In another study, the average tunnel length was evaluated as 32±5mm at <120° flexion, 36±4mm at 120° flexion, and 39±4mm at >130° flexion^6^. In our study, we evaluated the average tunnel length in group 1 on average 39.7 (30-45) mm, in group 2 on 36.7 (30-45) mm. Our tunnel length average was similar to the studies in the literature. Although the flexion was less in group 1, the average tunnel length was higher. We associate this with visualisation. It provides a clear observation of footprints and facilitates three angulations and three-dimensional orientation. Thanks to this, it allowed us to adjust the exit site in the lateral cortex of the tunnel. It provides spatially optimal interosseous tunnel placement. No patients were subjective complaints during follow-up. There were no patients who developed graft failure and rerupture. Also, the higher the degree of flexion, the lower the risk of damage to the lateral anatomical structures (especially to the peroneal nerve, lateral heads of the biceps, and gastrocnemius muscles). Chung *et al* reported that 14 (%23) patients could not perform more than 120° of hyperflexion in their 62 cases study^8^. In hyperflexion rymirization causes less damage to medial structures, especially the medial femoral condyle. As iatrogenic, 6 (15%) patients in Group 2 had chondral damage in MFC, while 1 (2.5%) patients in group 1 had chondral damage. No complication occurred during surgery except for chondral damage in MFC.

Many radiological measurements and surgical techniques have been identified for the correct anatomical femoral tunnel entry site. Some of these are the use of the midpoint of the anteromedial and posterolateral bundles, the use of a femoral offset guide, and fluoroscopic assisted tunnel formation. It has been reported that the application of the guidewire with an axial angle of 40° and forming a tunnel mouth of 136mm^2^ in the insertion area provides optimal tunnel placement^11,20,21,22^. We evaluated the femoral tunnel placement according to the Quadrant Method of Bernard and Hertel^5^. We evaluated (% Anterior-Posterior) 32 (26-38) in group 1, 32 (29-36) in group 2, 33 (27-37) in group 1 (% Proximal-Distal) and 32 (26-36) in group 2. Average measurements by Bernard and Hertel 24.8 / 28.5 (21), by Forsythe *et al*^21^, 28.4 / 44.25 (26) by Bird *et al*^14^ 27.3 / 34.35 it was evaluated as. In the study of Man Soo Kim *et al*^11^ the expert surgeon compared the quadrant method after ACLR performed by the novice surgeon. In the study, % proximal- distal; novice surgeon was reported as 30.5±4.6, expert surgeon 32.5±3.7, and in the anterior-posterior; novice surgeon 32.6±4.3, and expert surgeon 31.6±4.6. In this evaluation made according to the blumensaat line, it was stated that there was no difference in terms of both researchers. It is observed in the studies that there are important differences in radiological measurements. The fact that the studies have been applied single band or double band may explain the difference in radiological measurements. Similarly, we evaluated the axial tunnel angle as 39 (35-46) in group 1 and 38 (29-44)° in group 2. Kosy *et al*^6^ reported the axial tunnel angle as 40±6.8° in the flexible rymirization group and 37.4±7.5° in the rigid rymirization group. The axial tunnel angle is recommended to be 40° for optimal tunnel position. Our axial tunnel angle average was similar to the studies in the literature. The average axial tunnel angle in both groups was around 40°^21^. However, there was no difference between the mean axial tunnel angles statistically (p>.05).

Our study has some limitations. First, this study was a retrospective study. However, our records are kept regularly, and the data are checked from time to time and their accuracy confirmed. Secondly there was short follow-up time for graft failure and rupture evaluation. Therefore, there is a need for multicenter studies in which the mentioned factors are also evaluated, prospective, randomised controlled, long-term follow-up, more experienced surgeons are involved.

## Conclusion

Hyperflexion position is the most common method in femoral tunnel formation. However, our study shows that; during the reaming stage in the figure 4 position has less risk of damage to the medial femoral condyle. The position of Figure 4 provides visualisation of lateral intercondylar notch better than hyperflexion. With this feature, it provides longer femoral tunnel formation. In ACLR, which a long learning curve, short surgery time is seen as an important advantage for surgeons who have just started ACLR. We think that it can be used as an alternative method to hyperflexion in the learning curve process and maybe shorten the learning curve process.
